# Book Reviews

**Published:** 1904-11

**Authors:** 


### BOOK REVIEWS.

Radiotherapy and Phototherapy, including Radium and High Frequency Currents. Their Medical and Surgical Applications in Diagnosis and Treatment. By Charles Warrenne Allen, M.D., Professor of Dermatology in the New York Post-Graduate Medical School. Octavo, 618 pages, 131 engravings and 27 plates. Philadelphia and New York: Lea Brothers & Company. 1904. (Cloth, $4.50, net.)

Though the field of discussion covered by the foregoing title is comparatively new, yet already considerable, even a voluminous, literature,—monographic, journalistic, and textbookish,—has sprung up more or less suddenly. Some of it, to be sure, is of a studiously scientific order by thoughtful investigators who are trained to the work and know how to make deductions of a useful character from their experiments. Much of it, however, is of an ephemeral nature, written by those who have only the merest inkling of the details of radiology and radiotherapy, some of it even by men or women who are failures at every other kind of medicine.

We are led to these remarks, not because we desire to apply this criticism to the book before us, but rather because we take this opportunity of saying that, insofar as we may be able to determine, we shall exclude from our pages all references to *x*-ray and other like subjects that do not possess scientific value.

The author of this book appears to have made judicious selection of the literature he has examined, to which he has added his own observations based upon actual clinical and experimental work. We say "appears" because it is hardly possible in this uncertain field for even the most careful observer to determine at present the full value of his material. In an agreeable introduction Allen gives among other things a nomenclature which every physician would do well to commit to memory. A writer who does not understand the precise meaning of the technical terms used in discussing light and ray details is liable to bring confusion to his readers.

The work proper is divided into seven parts, in the first of which is given general considerations of radiology, including the history and character of the *x*-ray, the methods of its production apparatus, method of administration and other similar topics. The other parts consider respectively, diagnosis, radiotherapy, light, actinotherapy, radioactivity, and high-frequency currents. The latter subject is quite new to literature and it is probable that it will attract special attention for that reason.

It is an undoubted fact that many heretofore intractable diseases have become amenable to the influence of *x*-rays, these chiefly being affections of the skin, hence dermatologists must be depended upon to give us the best information concerning methods and results in this field. Nevertheless, there are a number of other diseases which have been treated with this mysterious agent with more or less success, according to the statements published. The list ranges all along the line from abscess of the antrum to splenic anemia, from epilepsy to tic-douloureux, from appendicitis to tuberculosis, and from neuralgia to paraplegia. Many of these diseases are mentioned by this author as having been treated with more or less success, and he is particularly replete with the use of the rays in diseases of the skin.

But taken all in all, perhaps the most interesting of his chapters are those relating to burns and other deleterious effects of the *x*-ray, because of the importance they bear to the entire subject. It is essential that we should know the dangers of a method,—quite as much so, indeed, as its benefits; ignorance here is unpardonable, but now, as always,"fools step in where angels fear to tread." The worker with the rays appear to be specially liable to suffer their deleterious effects, of which the well-known case of Edison's assistant is an example; and, sadly enough, another is that of a prominent orthopedic surgeon in Rochester who recently sustained amputation of his right hand and part of the left.

This book cannot fail to prove of interest to every worker with light and its collateral topics, and is a distinct addition to the literature of the subjects with which it treats.

A Handbook of Pathological Anatomy and Histology with an introductory section on Post-Mortem Examinations and the Methods of Preserving and Examining Diseased Tissues. By Francis Delafield, M.D., LL.D., Emeritus Professor of the Practice of Medicine, College of Physicians and Surgeons, Columbia University; and T. Mitchell Prudden, M.D., LL.D., Professor of Pathology and Director of the Department of Pathology, College of Physicians and Surgeons, Columbia University, New York. Seventh edition, with 13 full-page plates and 545 illustrations in the text, in black and colors. New York: William Wood & Company. 1904. (Price, $5.00.)

Since the previous edition of this work was issued Dr. Delafield has retired from the joint authorship that had existed from the beginning, hence Dr. Prudden is responsible alone for the alterations, additions, and revisions that are presented in this issue. For many years "Delafield and Prudden" has been the recognised authority on pathology and histology. Seldom has a treatise on these subjects been issued in so many successive editions and remained so long at the head of the list as a favorite both of teachers and students.

The revision of the text for the seventh edition has not been perfunctory; indeed, much of it has been rewritten and practically all of it has been revised,—certainly wherever revision was necessary, even the foot-notes having been included in this reconstruction. Particularly have the sections on the blood, malaria, and the nervous system received attention, while new material has been presented on immunity, Ehrlich's "side chain" hypothesis has been detailed, and cytolysis fully set forth.

The illustrations deserve special mention, many new plates and photographs having been added, making this the most completely illustrated work on pathology extant; besides, on this and various other accounts, it appears destined to occupy the center of the stage for a long time to come.

---

Diseases of the Nose and Throat. By D. Braden Kyle, M.D., Professor of Laryngology and Rhinology, Jefferson Medical College, Philadelphia; Consulting Laryngologist, Rhinologist and Otologist, Saint Agnes's Hospital. Third edition, thoroughly revised and enlarged. Octavo volume of 669 pages, with 175 illustrations, and 6 chromo-lithographic plates. Philadelphia, New York, London: W. B. Saunders & Company. 1904. (Cloth, $4.00 net; sheep or half morocco, $5.00 net.)

It is a genuine pleasure to note the frequent reappearance in revised editions of a good book like this one, for it must be admitted on all hands that it has fallen to the lot of Kyle to produce one of the really excellent medical works of the new century.

The author gives the following summary of the changes made in this issue: the general plan of the previous editions has not been materially altered. The entire book has been carefully revised and such additions have been made as were rendered necessary by recent medical progress. The most important alterations and additions have been made in the chapters on keratosis, epidemic influensa, Gersuny's paraffine method for the correction of nasal deformities, and in the one on the *x*-rays in the treatment of carcinoma. The etiology and treatment of hay fever have been partially rewritten and much enlarged, as has also the operative treatment of deformities of the nasal septum. In the chapter devoted to general considerations of mucous membranes and hay fever the author records the results of his experience in the chemistry of the saliva and nasal secretions in relation to diagnosis and treatment. The literature has been carefully reviewed, and a number of new illustrations added, thus bringing the work absolutely down to date.

The arrangement of the treatise is such as to make it easy of reference and in many ways it is specially adapted to the use of the general physician who, perforce, must deal with minor laryngology. We not only commend it to such, but to the specialist as well, who cannot afford to be without such a valuable contribution to the literature of the subject with which it deals.

---

A System of Practical Surgery. By Drs. E. von Bergmann, of Berlin, P. von Bruns, of Tübingen, and J. von Mikulicz, of Breslau. Edited by William T. Bull, M.D., Professor of Surgery in the College of Physicians and Surgeons (Columbia University), New York. To be complete in five imperial octavo volumes, containing over 4,000 pages, 1,600 engravings and 110 full-page plates in colors and monochrome. Volume III, 918 pages, 595 engravings, 21 plates. Sold by subscription only. New York and Philadelphia: Lea Brothers & Company. (Per volume, cloth, $6.00; leather, $7.00; half morocco, $8.50, net.)

The surgery of the extremities is dealt with in this splendid volume consisting of six sections, thirty-nine chapters, and nearly a thousand pages. The first section of seven chapters comprises the surgery of the shoulder and upper arm, for all their malformations, injuries and diseases; the second section of ten chapters treats of malformations, injuries and diseases of the elbow and forearm; the third section, in four chapters, handles malformations, injuries and diseases of the wrist and hand; the fourth section considers the surgery relating to malformations, injuries and diseases of the hip and thigh; the fifth section presents injuries and diseases of the knee and leg; and the sixth and final section offers the surgery pertaining to malformations, injuries and diseases of the ankle and foot.

This volume contains nearly six hundred illustrations,—drawings, photographs, wood engravings, and plates, some of the latter being in color. It forms an essential part of one of the most comprehensive and best exponents of the surgery of the present day that has been offered for professional favor and criticism.

---

Railway and other Accidents with Relation to Injury and Disease of the Nervous System. A Book for Court use. By Allan McLane Hamilton, M.D., F.R.S.E., New York. Octavo, 363 pages. Illustrated. New York: William Wood & Company. 1904. (Price, $3.50.)

It is quite unique to find a book, independently of works on medical jurisprudence, written for court use; but here is one intended for both doctors and lawyers when needed in the course of trial causes. Moreover, it is prepared by an expert in the witness box and an eminent specialist in diseases relating to the nervous system.

Railway spine, so-called, has become of late years the *bête noir* of courts lawyers and doctors. Many whose claims for damages were made on this account have secured favorable action,—justly some, unjustly others,—while a few deserving ones have been rejected. This, in a measure, is true of most court claims for railway damages, due in part to the incompetency of expert witnesses possibly, and in part to the "smart" lawyers probably; and in still other part to the laxity court administration. Whatever the cause or causes may be, this book if adquately studied by courts, lawyers, and doctors, will serve to remove, and hence will lead to a better understanding all around.

Contributory negligence, the stock plea of the railroads, often becomes a delicate question for consideration,—one of course in which the doctor has little to do; nevertheless, he should acquire a thorough understanding of its bearings in each particular case with which he deals.

Hamilton asseverates that medical truths are, in their way, quite as well settled as axioms of law. True, but in their interpretation lies the difficulty. This treatise will, *inter alia,* serve to expound them with a clearness that every conscientious court will appreciate and welcome as an aid in many of the difficult problems that are presented for solution. Lawyers and doctors will find it an interesting and instructive book.

---

The Practical Medicine Series of Year Books. Ten volumes. Issued under the general editorial charge of Gustavus P. Head, M.D., Professor of Laryngology and Rhinology in the Chicago Post-Graduate Medical School. Volume VI. General Medicine. Edited by Frank Billings, M.D., and J. H. Salisbury, M.D., Chicago. Duodecimo, pp. 330. Chicago: The Year Book Publishers. 1903. (Price, $1.00; entire series, $5.50, payable in advance.)

The subjects dealt with under the head of general medicine are, perforce, of a practical character. The clinician must know the symptomatology, pathology and management of typhoid fever, and he will find the latest thought relating to this disease embodied in this number of the Year Books. For example, the anomalies of the disease are dealt with by Elsner; the complications, by Osler; relapses and post-typhoid manifestations, by Delafield. Diet is well considered by le Fevre; hydrotherapy, by Glentworth R. Butler. The classic types of the disease are well understood; it is the anomalies, the aberrant forms, that attract attention at the bedside; hence the value of just such works as this.

Much of the volume is devoted to diseases of the gastro-intestinal tract and all the later methods of treatment are set forth.

---

The Clinical Study of Blood-pressure. A Guide to the Use of the Sphygmomanometer. By Theodore C. Janeway, M.D., Lecturer on Medical Diagnosis, University and Bellevue Hospital Medical College, New York. Small octavo, pp. 313. Illustrated. New York and London: D. Appleton & Company. 1904. (Price, $3.00.)

It is inadequate nowadays to say that a patient has fever; we must be able to record the precise registration of the thermometer in degrees and fractions, else we may be charged with carelessness or even worse,—neglectfulness. That is what the clinical thermometer is for—namely, precision as against guess-work. So, too, in regard to blood-pressure; the sphygmomanometer is designed to tell us its precise volume, instead of permitting us to be satisfied with the mere impression the radial pulse conveys to the finger tips.

Janeway, the younger, has been the first to present the profession with a detailed statement in book form of the methods of measuring blood-pressure. It is not to be expected that all the facts are in at present, but sufficient have been established to justify the publication of this monograph, which may be regarded as a guide to the use of the sphygmomanometer and other instruments employed in measuring blood-pressure. It contains a description of these several devices, gives their cost, and mentions where they may be purchased.

The book discusses the physiology and pathology of blood-pressure in the full light of our present knowledge, the state of arterial pressure in different diseases is given, and the entire topic is dealt with in its relation to medicine, surgery, and obstetrics; diagnosis, prognosis, and therapeutic indications being given deserved consideration in all their special bearings. It is a work likely to attract the attention of all clinicians and laboratory workers, and should find its way into the hands of all physicians who are concerned in accuracy of diagnosis.

---

The Utero-ovarian Artery. Anatomy and Physiology with their Application in Diagnosis and Surgical Intervention. By Byron Robinson, M.D., Chicago. E. H. Colegrove, Chicago. 1903. (Price, $1.00.)

Studies in anatomy relating to special regions, are always of value in clearing away doubtful points of pathology or in aiding in the solution of difficult problems in surgery. In this particular instance the abdominal and pelvic surgeon will be benefited by carefully examining the drawings, plates, and text, as they serve to elucidate many intricacies of artery distribution and ureteral relationship.

It is a work that represents vast industry and no end of patience,—a labor that has extended through years of research. We could wish that it had been printed in better form, or at least that the ink had been more evenly spread. Such painstaking dissections, injections, methods in corrosion, anatomy and other features deserve the best portrayal the printer's art can give.

---

Medical and Surgical Report of the Presbyterian Hospital, New York City. Volume VI., January, 1904. Edited by Andrew J. McCosh, M.D., and W. Gilman Thompson, M.D.

As usual with these reports the one before us comes freighted with excellent material. It is prepared with the same scrupulous care that is given to an ordinary textbook and is printed on a finished paper that makes the half-tone illustrations show exceeding well; moreover, the careful English of the writers bespeaks interest in their work. Some of the articles,—for example, that on "septic peritonitis from appendicitis,"—will be referred to as authoratative. It is a genuine pleasure to receive such a hospital report as this.

